# Assessing worldwide research activity on probiotics in pediatrics using Scopus database: 1994–2014

**DOI:** 10.1186/s40413-016-0116-1

**Published:** 2016-07-25

**Authors:** Waleed M. Sweileh, Naser Y. Shraim, Samah W. Al-Jabi, Ansam F. Sawalha, Belal Rahhal, Rasha A. Khayyat, Sa’ed H. Zyoud

**Affiliations:** 1 Department of Pharmacology and Toxicology, College of Medicine and Health Sciences, An-Najah National University, Nablus, 44839 Palestine; 2 Department of Pharmaceutical Chemistry and Technology, College of Medicine and Health Sciences, An-Najah National University, Nablus, 44839 Palestine; 3 Department of Clinical and Community Pharmacy, College of Medicine and Health Sciences, An-Najah National University, Nablus, 44839 Palestine; 4 Department of Biomedical Sciences, Faculty of Medicine and Health Sciences, An-Najah National University, Nablus, 44839 Palestine; 5 Division of Microbiology and Immunology, Department of Bio-Medical Sciences, College of Medicine and Health Sciences, An-Najah National University, Nablus, 44839 Palestine

**Keywords:** Pediatrics, Probiotics, Bibliometric analysis, Scopus

## Abstract

**Background:**

A wide variety of probiotic products has been introduced into the market in the past decade. Research trends and activity on probiotics help understand how these products were evolved and their potential future role in medicine. The objective of this study was to assess the research activity on probiotics in pediatrics using bibliometric indicators and network visualization.

**Methods:**

Original and review articles on probiotics in pediatrics published worldwide were retrieved from SciVerse, Scopus (1994–2014) and analyzed. VOSviewer was used for network visualization.

**Results:**

The total number of documents published on probiotics in pediatrics was 2817. Research activity on probiotics in pediatrics showed approximately 90- fold increase during the study period. Approximately 22 % of published articles originated from USA and has the greatest share, however, Finland ranked first when data were stratified by population or income. The most productive institution in this field was Turku University in Finland with 82 (2.91 %) articles. Half of the prolific authors were also from Finland. Most of the published research activity appeared in *Journal of Pediatric Gastroenterology and Nutrition*. Most frequently encountered title terms include nutrition, infant formula, necrotizing enetrocolitis, allergy, and diarrhea. The total number of citations for the retreived documents documents was 70991, and the average citation per article was 25.20.

**Conclusions:**

Interest in probiotic research and its potential benefits in pediatric ailments is relatively recent but significantly increasing. Bibliometric analysis can be used as an indicator of the importance and growth of probiotic use in pediatrics.

## Background

Probiotics are defined as live microbes which can benefit the host when consumed in sufficient amounts [1, 2]. Probiotics have been described as friendly bacteria with host benefits [3]. Common probiotics include species with the genera *Lactobacillus* and *Bifidobacteria* which can be found in some commercial dairy products and cereals [1, 2]. Probiotic products are available in the market and interest of consumers and clinicians in these products is growing as evident by the tremendous increase in the sale of these products over a short period of time [4, 5]. The friendly nature of these products and their classification as dietary supplements has increased the popularity and marketability of these products. The potential benefits of the gut microbiota on immune function encouraged researchers to investigate potential health effects of gut microbiota [6]. Furthermore, the diversity of health conditions claimed to be treated by probiotics made clinicians and researchers keen to investigate and learn more about these relatively safe and natural products. Some conditions that probiotics might benefit include inflammatory bowel disease (IBD), irritable bowel syndrome (IBS), antibiotic-associated diarrhea (AAD), necrotizing enterocolitis (NEC), and other conditions [7–9]. Better understanding of research trends in any medical field requires a full understanding of the research activity in that field and the key researchers and institutes in that particular field. Such understanding of the research activity could be achieved through bibliometric analysis using well known databases. In the case of probiotics, and up to the author’s best knowledge, no bibliometric studies on probiotics in pediatrics have been published. Therefore, we sought to assess trends in research activity about probiotics in pediatrics. This is important for future comparative analysis on probiotics research. Such bibliometric and comparative information are important for clinicians, health policy makers, consumers and manufacturers given that safety and efficacy of probiotics for health problems in infants and children are not yet well established.

## Methods

The data in this study were synthesized using Scopus database which has many important features that facilitate bibliometric analysis as in previous similar studies [10–15]. Such features include citation analysis, country and author contribution as well as source titles and productivity per year. Scopus is produced by Elsevier and covers more than 20,000 journals that have 100 % Medline coverage. Scopus is larger than Web of Science and more accurate than Google Scholar [16].

The study period was set from January 01, 1994 to December 31, 2014. All subject areas in Scopus search engine (life sciences, social sciences, health and physical sciences) were chosen. The date for the study was set up to 2014 because data for 2015 and 2016 are not yet complete in Scopus since some journal may require 1–2 years to transfer its content to databases. Therefore, inclusion of 2015 and 2016 will create some inaccuracy problems. The search strategy in this article was based on retrieving articles with the keyword probiotics in title-abstract-key and keywords relevant to the term “pediatrics” in article title or keywords. All articles with the keyword “adult” were excluded. To increase the accuracy of our search, documents classified as errata, or books, or book chapter or un-defined type of documents were excluded and therefore this study is restricted to documents that are considered journal articles. All documents obtained after refining the results were transferred to Statistical Package for Social Sciences software version 20 to present the bibliometric indicators. The validity of our method was assessed by assessing the top 300 (~10 % of the results) cited documents retrieved by the method mentioned above to assure that the content of these retrieved articles matches the search query of interest.

The main bibliometric indicators presented in this study included type and language of the published documents, country and institutional affiliation, source/ journal title in which documents were published, most productive authors, most cited articles, and collaboration patterns. Many of the bibliometric indicators were presented in rank order. Research productivity was assessed by the quantity of publications while the total number of citations was used to identify the most influential articles in the field [17–22]. The impact factor (IF) of journals was used as a measure of quality of journals and was obtained from Thompson Reuters [23]. The Hirsch index (h-index) was used to assess the quantity and quality of publications per country or per institution or per author [24]. The research productivity of different countries was normalized using population size and national Gross Domestic Product (GDP) retrieved from the online databases of the World Bank [25].

Bibliometric maps and network visualization methods were made using VOSviewer software [26]. Using the VOSviewer and thresholds of minimally ten fractionally counted articles for each term, density visualization maps were generated for most frequently encountered terms in title of retrieved articles. In these maps, most frequent terms had dense colored cluster. For co-authorship analysis, a minimum number of 500 authors were selected in VOSviewer program. Authors located within or close to a large cluster are believed to have higher number of co-authors suggestive of inter and intra country collaboration.

The methodology used in this study was similar to recent bibliometric studies published by the same research group using SciVerse Scopus [27–32]. All data and documents were extracted and analyzed on 12^th^ of April, 2016. Since the data for this study was obtained from electronic sources that are publicly available and not pertaining to specific patients’ data or profile, IRB ethical approval for the study was not required.

## Results

### General data

Based on the search strategy implemented, a total of 2817 articles were retrieved. Around half (50.76 %) of these articles were original research (Table 1). The total number of different languages encountered in the retrieved articles was 25 and the primary language was English (2416, 85.76 %). Other encounterd languages like Spanish, German, French, Polish Russian, Italian, Chinese Czech and Dutch are shown in Table 2.

**Table 1:** Number and percentage of each type of published articles on probiotics in pediatrics (1994–2014)

Type of document	Number (%)
Article	1430 (50.76)
Review	822 (29.18)
Conference papers	172 (6.11)
Note	122 (4.33)
Editorial	98 (3.48)
Letter	98 (3.48)
Short survey	63 (2.24)
Article in Press	12 (0.43)

**Table 2:** Top 10 languages encountered in retrieved articles on probiotics in pediatrics (1994–2014)

Rank	Language	Frequency (%)
1	English	2416 (85.76)
2	Spanish	72 (2.56)
3	German	71 (2.52)
4	French	68 (2.41)
5	Polish	58 (2.06)
6	Russian	37 (1.31)
7	Italian	34 (1.21)
8	Chinese	26 (0.92)
9	Czech	11 (0.39)
10	Dutch	9 (0.32)

The total number of citations for the retrieved articles was 70991 and the average citation per article was 25.20. A total of 610 (21.65 %) retrieved articles were not cited while 2207 (78.35 %) were cited at least once. As expected, the zero citation was highest for articles published in 2014 compared to ones published in previous years. The number of articles which received at least 50 citations was 364 articles (12.92 %).

### Most frequent terms

**Fig. 1:**
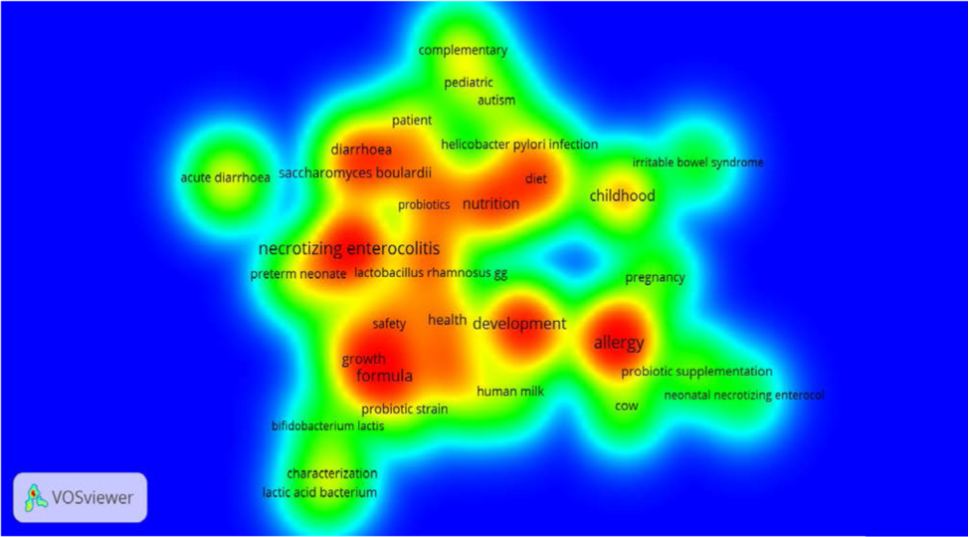
Map of frequently encountered title terms in retrieved articles on probiotics in pediatrics

In mapping terms frequency network, from the 3737 terms, 112 terms met the threshold of ten times as a minimum number of occurrences. Then 56 terms were selected as relevant terms based on calculated relevance score. Figure 1 shows the visualization network map of most frequently encountered terms in the title of retrieved articles. Based on the map, seven clusters were located: cluster number one contained 11 terms with the term nutrition as most frequent one; cluster number two contained nine items with the terms formula/ infant formula being most frequent; cluster number three contained eight items with the term necrotizing enterocolitis being most frequently encountered; cluster number four contained eight items with the term enterocolitis being most frequently encountered; cluster number five contained eight items with term allergy being most frequently encountered; cluster number six contained eight items with the term childhood being most frequently encountered; and finally cluster number seven contained eight items with the term diarrhea being most frequently encountered (Fig. 1).

### Publications with time

**Fig. 2:**
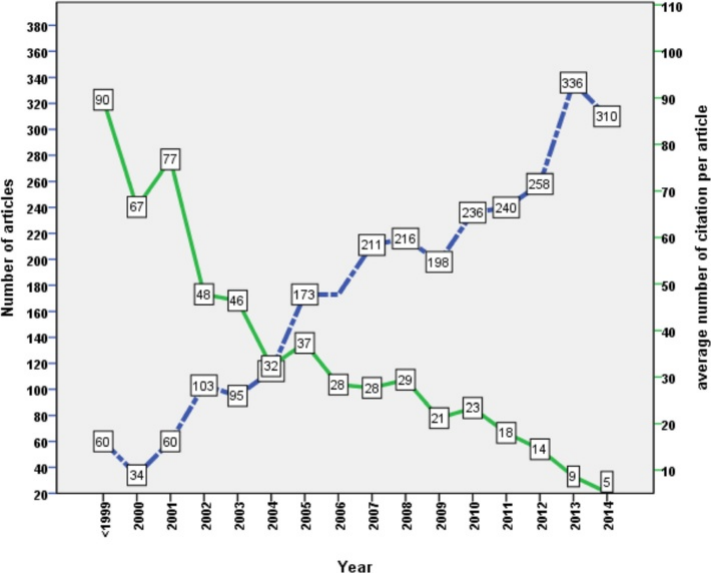
Evolution of the number of published articles and citations per article

The majority of retrieved articles (2351; 83.46 %) were published in the past decade (2005–2014). Table 3 shows the number of retrieved articles per year. Figure 2 shows the average citations per article and the number of published articles over the study period. Articles published before year 2000 had the highest average citations per article.

**Table 3:** Number of published articles and citations on probiotics in pediatrics

Year	Number of documents *N* = 2817 (%)	Total citation	Citations/article
1994–1999	60 (2.13)	5376	89.60
2000	34 (1.21)	2263	66.56
2001	60 (2.13)	4605	76.75
2002	103 (3.66)	4922	47.79
2003	95 (3.37)	4412	46.44
2004	114 (4.05)	3686	32.33
2005	173 (6.14)	6455	37.31
2006	173 (6.14)	4906	28.36
2007	211 (7.49)	4836	27.66
2008	216 (7.67)	6355	29.42
2009	198 (7.03)	4198	21.20
2010	236 (8.38)	5514	23.36
2011	240 (8.52)	4324	18.02
2012	258 (9.16)	3738	14.49
2013	336 (11.93)	2901	8.63
2014	310 (11.00)	1607	5.18

### Countries

Table 4 shows the top ten productive countries from a total of 102 countries which contributed to the advancement of research on probiotics in pediatrics. The USA had the greatest share of publications was also the leading country in the annual number of publications. Publications from USA had the highest share of citations (18665), however, publications from Finland had the highest number of citations per article (87.10). When countries were ranked based on the h-index of their research activity on probiotics in pediatrics, USA ranked first (69) followed by Italy (48) and Finland (46) (Table 4).

International (inter country) collaboration was also shown in Table 4. Articles from USA (25.00 %) and Poland (21.99 %) had the least percentage of inter-country collaboration. For USA and Poland, more than 75 % of articles were published by domestic authors. On the other hand, more than half (54.63 %) of articles from Netherlands, for example, had co-authors from other different countries. For the top 10 productive countries a total of 629 (31.91 %) articles had multiple country affiliations while the remaining (68.09 %) published articles were publications from single country collaboration.

**Table 4:** Top 10 productive countries in number of probiotics in pediatrics publications

SCR^a^	Country	Articles *N* = 2817 (%)	Articles/10 million inhabitants (Rank)	Articles /trillion GDP (Rank)	Total citation (Rank)	Citations/article (Rank)	H-index (Rank)	Number of Collaborating countries	Number (%)^b^ of documents with international authors
1^st^	USA	616 (22.36)	19.32 (9)	35.36 (10)	18665 (1)	30.30 (6)	69 (1)	61	154 (25.00)
2^nd^	Italy	265 (8.98)	43.2 (4)	123.77 (3)	8093 (3)	30.54 (5)	48 (2)	45	81 (30.57)
3^rd^	United Kingdom	167 (6.26)	25.89 (7)	55.87 (7)	7409 (4)	44.37 (3)	45 (4)	52	66 (39.52)
4^th^	Germany	155 (5.14)	19.16 (10)	40.07 (9)	4982 (6)	32.14 (4)	42 (5)	43	67 (43.23)
5^th^	Poland	141 (5.00)	37.11 (5)	258.72 (2)	3664 (7)	25.99 (9)	29 (9)	29	31 (21.99)
6^th^	Finland	136 (4.86)	248.90 (1)	499.63 (1)	11845 (2)	87.10 (1)	46 (3)	38	44 (32.35)
7^th^	France	132 (4.48)	19.94 (8)	46.66 (8)	3622 (8)	27.44 (8)	30 (7)	40	50 (37.88)
7^th^	Spain	132 (4.48)	28.45 (6)	95.58 (5)	2537 (10)	19.20 (10)	25 (10)	37	38 (28.88)
9^th^	Australia	119 (4.34)	50.66 (3)	81.79 (6)	3333 (9)	28.01 (7)	30 (7)	40	39 (32.77)
10^th^	Netherlands	108 (4.26)	64.09 (2)	122.82 (4)	5212 (5)	48.26 (2)	37 (6)	45	59 (54.63)

*Abbreviations*: *SCR* standard competition ranking, *USA* United States of America, *GDP* gross domestic product

^a^Equal countries have the same ranking number, and then a gap is left in the ranking numbers

^b^Percentage of documents with international authors from the total number of documents for each country

### Authors

**Fig. 3:**
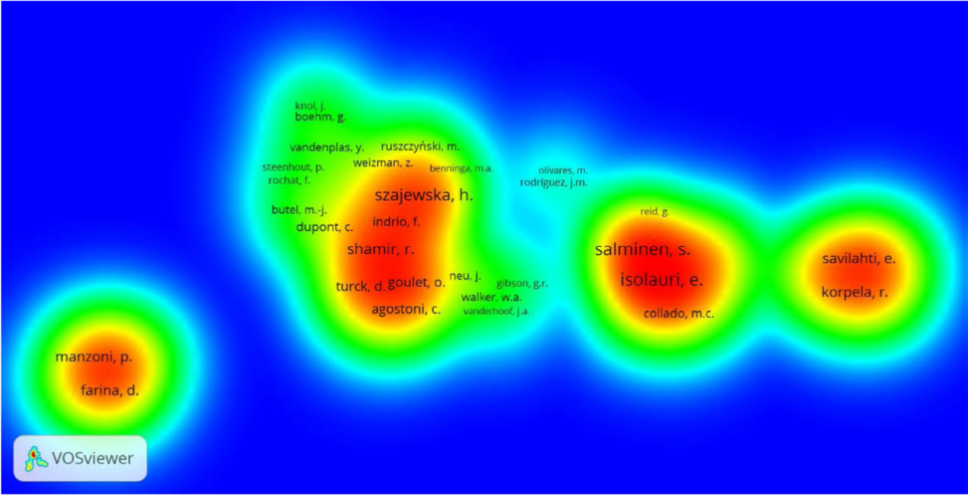
Co-authorship analysis for retrieved articles on probiotics in pediatric research (1994–2014)

Professor Isolauri, E. from Finland and Professor Szajewska, H. from Poland ranked first in the number of publications with 68 (2.41 %) articles for each (Table 5). Besides, Isolauri, E. ranked first in h-index. Of the most prolific authors, five were from Finland, two were from the USA, one from Poland, one from Belgium and one from Italy. Density visualization of co-authorships using authors as unit of analysis showed that co-authorships were high and common among most prolific authors (Fig. 3). Co-authorships is suggestive of domestic and international collaboration. Authors who were remotely located from clusters have relatively fewer co-authorships and collaborations. In density visualization map, out of 7589 authors, 51 met the threshold of ten and out of 51, 43 were selected based on relevance score. The map contained eight clusters: cluster number one contained ten authors with Vanderplas, Y. having highest number of co-authorships; cluster number two contained seven items with Isoluri, E., Kalliomaki, M., and Salmenin, S. having highest number of co-authorships; cluster number three which contained 6 items with Shamir, I. having highest number of co-authorships; cluster number four contained five authors with Savilhati, E., Kuitunen, M., and Korpela, R. having the highest number of co-authorships; cluster number five contained authors with Szajewska, H. and Guarino, A. having the highest number of co-authorships; cluster number six with four authors with Manzoni, P. having the highest number of co-authorships; cluster number seven contained four authors with Neu, J. and Walker, W. having the highest number of co-authorships; and finally cluster number eight contained two authors with Rodriguez, J. having highest number of co-authorships.

**Table 5:** Top 10 authors publishing on probiotics in pediatrics

Rank (R)	Author	Number of published articles	Cluster (number of co-authorships)	Total citation (R)	h-index (R)	Country
1	Isolauri, E.	68	2 (82)	8873 (1)	35 (1)	Finland
1	Szajewska, H.	68	5 (67)	3164 (4)	28 (3)	Poland
3	Salminen, S.	53	2 (73)	7325 (2)	29 (2)	Finland
4	Vandenplas, Y.	38	1 (10)	681 (10)	16 (6)	Belgium
5	Neu, J.	29	7 (13)	1476 (7)	18 (4)	USA
6	Walker, W.A.	26	7 (7)	1626 (6)	18 (4)	USA
7	Guarino, A.	24	5 (16)	1064 (8)	14 (8)	Italy
8	Korpela, R.	22	4 (33)	1662 (5)	13 (9)	Finland
9	Kalliomaki, M.	21	2 (38)	3837 (3)	16 (6)	Finland
10	Kuitunen, M.	19	4 (31)	1064 (8)	12 (10)	Finland

*R* rank

### Frequently cited articles

The top 10 cited articles about probiotics in pediatrics are presented in Table 6 [33–42], Table 7 [43–52] and Table 8 [53–62] based on year intervals. The article which received the highest citation was “*Probiotics in primary prevention of atopic disease: A randomised placebo-controlled trial*” authored by Kalliomäki et al. and published in 2001 in *Lancet* journal. The article received a total of 1696 citations up to the time of analysis of data (April 12, 2016).

**Table 6:** Top 10 cited articles on probiotics in pediatrics (1994–2000) [33–42]

SCR	Authors	Title	Year	Source title	Cited by
1^st^	Majamaa & Isolauri [39]	Probiotics: A novel approach in the management of food allergy	1997	*Journal of Allergy and Clinical Immunology*	659
2^nd^	Isolauri et al. [38]	Probiotics in the management of atopic eczema	2000	*Clinical and Experimental Allergy*	638
3^rd^	Björkstén et al. [34]	The intestinal microflora in allergic Estonian and Swedish 2-year-old children	1999	*Clinical and Experimental Allergy*	624
4^th^	Collins & Gibson [35]	Probiotics, prebiotics, and synbiotics: Approaches for modulating the microbial ecology of the gut	1999	*American Journal of Clinical Nutrition*	502
5^th^	Guandalini et al. [36]	Lactobacillus GG administered in oral rehydration solution to children with acute diarrhea: A multicenter European trial	2000	*Journal of Pediatric Gastroenterology and Nutrition*	443
6^th^	Vanderhoof et al. [42]	Lactobacillus GG in the prevention of antibiotic-associated diarrhea in children	1999	*Journal of Pediatrics*	358
7^th^	Salminen [41]	Clinical uses of probiotics for stabilizing the gut mucosal barrier: Successful strains and future challenges	1996	*Antonie van Leeuwenhoek, International Journal of General and Molecular Microbiology*	293
8^th^	Arvola et al. [33]	Prophylactic Lactobacillus GG reduces antibiotic-associated diarrhea in children with respiratory infections: a randomized study.	1999	*Pediatrics*	279
9^th^	Pessi et al. [40]	Interleukin-10 generation in atopic children following oral lactobacillus rhamnosus GG	2000	*Clinical and Experimental Allergy*	273
10^th^	Hoyos [37]	Reduced incidence of necrotizing enterocolitis associated with enteral administration of Lactobacillus acidophilus and Bifidobacterium infantis to neonates in an intensive care unit	1999	*International Journal of Infectious Diseases*	231

*SCR* standard competition ranking

**Table 7:** Top 10 cited articles on probiotics in pediatrics (2001–2007) [43–52]

SCR	Authors	Title	Year	Source title	Cited by
1^st^	Kalliomäki et al. [46]	Probiotics in primary prevention of atopic disease: A randomised placebo-controlled trial	2001	*Lancet*	1696
2^nd^	Kalliomäki et al. [47]	Probiotics and prevention of atopic disease: 4-year follow-up of a randomised placebo-controlled trial	2003	*Lancet*	825
3^rd^	Ouwehand et al. [50]	Probiotics: An overview of beneficial effects	2002	*Antonie van Leeuwenhoek, International Journal of General and Molecular Microbiology*	518
4^th^	Isolauri et al. [45]	Probiotics: Effects on immunity	2001	*American Journal of Clinical Nutrition*	495
5^th^	D’Souza et al. [43]	Probiotics in prevention of antibiotic associated diarrhoea: Meta-analysis	2002	*British Medical Journal*	463
6^th^	Van Niel et al. [52]	Lactobacillus therapy for acute infectious diarrhea in children: A meta-analysis	2002	*Pediatrics*	413
7^th^	Lin et al. [48]	Oral probiotics reduce the incidence and severity of necrotizing enterocolitis in very low birth weight infants	2005	*Pediatrics*	401
8^th^	Lin & Stoll [49]	Necrotising enterocolitis	2006	*Lancet*	396
9^th^	Rosenfeld et al. [51]	Effect of probiotic Lactobacillus strains in children with atopic dermatitis	2003	*Journal of Allergy and Clinical Immunology*	376
10^th^	Hatakka et al. [44]	Effect of long term consumption of probiotic milk on infections in children attending day care centres: Double blind, randomised trial	2001	*British Medical Journal*	366

*SCR* standard competition ranking

**Table 8:** Top 10 cited articles on probiotics in pediatrics (2008–2014) [53–62]

SCR	Authors	Title	Year	Source title	Cited by
1^st^	Neu & Walker [61]	Necrotizing enterocolitis	2011	*New England Journal of Medicine*	412
2^nd^	Van Assche et al. [62]	The second European evidence-based Consensus on the diagnosis and management of Crohn’s disease: Special situations	2010	*Journal of Crohn’s and Colitis*	382
3^rd^	Kalliomäki et al. [57]	Early differences in fecal microbiota composition in children may predict overweight	2008	*American Journal of Clinical Nutrition*	340
4^th^	Agostoni et al. [53]	Enteral nutrient supply for preterm infants: Commentary from the european society of paediatric gastroenterology, hepatology and nutrition committee on nutrition	2010	*Journal of Pediatric Gastroenterology and Nutrition*	328
5^th^	Macfarlane et al. [59]	Bacterial metabolism and health-related effects of galacto-oligosaccharides and other prebiotics	2008	*Journal of Applied Microbiology*	283
6^th^	Deshpande et al. [55]	Updated meta-analysis of probiotics for preventing necrotizing enterocolitis in preterm neonates	2010	*Pediatrics*	274
7^th^	Hsiao et al. [56]	Microbiota modulate behavioral and physiological abnormalities associated with neurodevelopmental disorders	2013	*Cell*	269
8^th^	Maslowski & MacKay [60]	Diet, gut microbiota and immune responses	2011	*Nature Immunology*	261
9^th^	Lin et al. [58]	Oral probiotics prevent necrotizing enterocolitis in very low birth weight preterm infants: A Multicenter, Randomized, Controlled trial	2008	*Pediatrics*	235
10^th^	Arslanoglu et al. [54]	Early dietary intervention with a mixture of prebiotic oligosaccharides reduces the incidence of allergic manifestations and infections during the first 2 years of life	2008	*Journal of Nutrition*	227

*SCR* standard competition ranking

### Institutions

The most research productive institution was Turun yliopisto (i.e., University of Turku) in Finland with 82 (2.91 %) publications and an h-index of 34. Furthermore, Turun yliopisto also ranked first in total citations 8095 (Table 9).

**Table 9:** Top 10 list of institutions that published research articles on probiotics in pediatrics (1994–2015)

SCR^a^	Institution	Number of documents *N* = 2817 (%)	Total citation (Rank)	Citations/article (Rank)	H-index (Rank)	Affiliation country
1^st^	*Turun yliopisto*	82 (2.91)	8095 (1)	98.72 (2)	34 (1)	Finland
2^nd^	*Medical University Warsaw*	72 (2.56)	3234 (3)	44.92 (5)	27 (2)	Poland
3^rd^	*Universita degli Studi di Napoli Federico II*	45 (1.60)	1805 (6)	40.11 (8)	23 (3)	Italy
4^th^	*Universita degli Studi di Milano*	44 (1.56)	1864 (5)	42.36 (7)	19 (6)	Italy
5^th^	*Massachusetts General Hospital*	37 (1.31)	2105 (4)	56.89 (3)	22 (4)	USA
6^th^	*Turun Yliopistollinen Keskussairaala*	36 (1.28)	5103 (2)	141.75 (1)	21 (5)	Finland
7^th^	*Universite Paris Descartes*	35 (1.24)	1553 (7)	44.37 (6)	18 (7)	France
8^th^	*Nestle*	32 (1.14)	1072 (9)	33.50 (9)	17 (8)	Switzerland
9^th^	*University of Florida*	29 (1.03)	1410 (8)	48.62 (4)	16 (9)	USA
9^th^	*Universitair Ziekenhuis Brussel*	29 (1.03)	567 (10)	19.55 (10)	14 (10)	Belgium

*SCR* standard competition ranking, *USA* United States of America

^a^Equal institutions have the same ranking number, and then a gap is left in the ranking numbers

### Journals

The retrieved articles were published in 151 different journal names. A total of 454 (16.12 %) articles were published in the top ten productive journals (Table 10). The journal that has the largest share of publications was *Journal of Pediatric Gastroenterology and Nutrition* (*n* = 127). The journal that received the greatest number of citations was also the *Journal of Pediatric Gastroenterology and Nutrition*. However the of citations per article was greatest for *Journal of Allergy and Clinical Immunology* 112.59) followed by *Clinical and Experimental Allergy* journal (80.44). Table 10 also shows the impact factor values for the top ten productive journals.

**Table 10:** Top 10 list of journals in which research documents on probiotics in pediatrics were published

SCR^a^	Journal	Number of articles (%)	Total number of citations	Number of citations per article	h-index	Impact Factor (Rank)
1^st^	*Journal of Pediatric Gastroenterology and Nutrition*	127 (4.51)	5496	43.27	42	2.625 (7)
2^nd^	*Pediatrics*	61 (2.17)	4055	66.47	29	N/A (10)
3^rd^	*Journal of Pediatrics*	50 (1.77)	2477	49.54	21	3.790 (3)
4^th^	*Pediatric Allergy and Immunology*	39 (1.38	978	25.08	19	3.397 (6)
5^th^	*British Journal of Nutrition*	37 (1.31)	1552	41.95	20	3.453 (5)
6^th^	*Journal of Clinical Gastroenterology*	33 (1.17)	857	25.97	17	3.498 (4)
7^th^	*Clinical and Experimental Allergy*	32 (1.14)	2574	80.44	19	4.769 (2)
8^th^	*Journal of Allergy and Clinical Immunology*	27 (0.96)	3040	112.59	19	11.476 (1)
9^th^	*Pediatria Wspolczesna*	24 (0.85)	47	1.96	4	0.00 (9)
9^th^	*Early Human Development*	24 (0.85)	505	21.04	14	1.785 (8)

*SCR* standard competition ranking, *N/A* not available

^a^Equal journals have the same ranking number, and then a gap is left in the ranking numbers

## Discussion

In the current study, we focused on 2817 published articles on probiotic in pediatrics. These articles were retrieved using Scopus database which is a large and trustful database. All articles about probiotics published in other databases like pubmed are also found in Scopus. Therefore, using other databases will not change the results. However articles about probiotics that are not indexed in any database such as articles published in local journal in developing countries that are not indexed in Scopus could have been missed. Overall, we consider the results obtained are accurate and valid since manual review of 10 % of the top cited articles showed that all articles were on probiotics in pediatrics.

Although the history on probiotics research goes back to the early 20th century [63], scientific research on probiotics in pediatrics was first published in 1993 [64, 65]. Evidence – based management of pediatric illnesses such as diarrhea, allergy and other ailments requires an understanding of how scientific research about various medications has progressed. One method to assess past and current status of medications is to assess research output for that particular medication. In case of probiotics, the number of clinical trials investigating both the efficacy and safety of probiotic products has increased exponentially with great evidence of safety but varying degrees of efficacy depending on the strain and medical condition being treated [3, 8, 66].

The number of published articles in this field has increased by greater than 90-fold during the past 20 years. The growth in probiotic research is accompanied by an overall increase in various medical and biomedical fields, an increase in the number of journals particularly in the field of pediatrics, and finally by the advancement in microbiology. The presence of articles with non-English language is also an indicator of worldwide growing interest in probiotics. The noticable increase in publications suggests that there is a large general audience for probiotics as a means of therapy for pediatric aliments. The fact that non-academic institutions ranked among top 10 in probiotic research indicated that this topic is a very interesting issue from a clinical, nutritional, econmic and consumer aspects. In addition, research about probiotics and its potential association with enhancing immunity and combating allergy and asthma gave further momentum to probiotic research in pediatrics [47, 67, 68].

Our results indicated that probiotics have been investigated as a potential therapy to prevent and treat a wide variety of pediatric ailments; mainly diarrhea, allergy, and gastrointestinal problems such as infantile colic, functional constipation, irritable bowel syndrome, ulcerative colitis, necrotizing enterocolitis and prevention of dental caries. Based on a recent literature review study, the most common health applications for probiotics include diarrhea, prevention of allergies, and treatment of a wide range of bowel diseases [3]. There is a general agreement that probiotics are modestly effective in treatment or prevention of acute infectious diarrhea [69, 70] but are of a significant benefit in decreasing incidence of antibiotic-associated diarrhea [71]. However, there is still inadequate evidence to support routine use of probiotics in the prevention of antibiotic-associated *Clostridium difficile* infectious diarrhea or non-*Clostridium difficile* antibiotic associated diarrhea [72, 73]. Studies regarding use of probiotics in prevention or treatment of eczema showed conflicting results and therefore, probiotics are not recommended for routine use of eczema [1]. Probiotics are currently marketed as dietary supplements and in infant formulas and dairy foods such as yogurt and therefore no FDA approval is needed for their marketing. However, other probiotic products such as those used in treatment and/or prevention of a disease, are considered biologic products, and are extensively reviewed and regulated by the FDA [74].

Analysis of countries involved in probiotic research related to pediatric therapies showed that the USA was dominant in this field. This was not surprising given that the USA ranked first in most worldwide bibliometric analysis of various medical fields [75–77]. The sales of probiotics in the USA grew by 31 % during 1 year and it is expected that annual sales to reach $31.1 billion by 2015 and might even be better depending upon consumer’s education and awareness of the safety and value of these products [78]. The majority of top ranking countries and institutions were European, probably due to advanced infant food industries in those particular European countries. Turun yliopisto (i.e., University of Turku) in Finland had the greatest share of publications, total citations and h-index value while *Turun Yliopistollinen Keskussairaala* (Hospital District of Southwest Finland) ranked first in average number of citations per article. Both institutions are in Finland. Furthermore half of the top prolific authors on probiotics in pediatrics are from Finland. All this made Finland to rank number one country in research activity on probiotics in pediatrics when measured per population or per GDP. Unfortunately, none of the top ten countries or institutions were from Latin America or Africa, or Middle East.

Publications from Finland showed dominant domestic collaboration with one third being from international collaboration which is lesser than that in UK or Germany but higher than that in USA. International collaboration in probiotic research and publication should be encouraged and emphasized given that probiotics could be of great value to children in developing countries were expensive medications might not be available. Furthermore, collaboration increases the probability of citations independent of time since publication, journal, or the country of the author [79].

The top 10 cited articles over the past two decades revealed that most of the hot articles were focused on the role of probiotics in treatment of allergy and diarrhea in children. The other ones were related to microbiology, immunology and potential mechanism of benefits of probiotics in children. No wonder that such hot articles were mainly published in highly prestigious journals in the field of allergy/ immunology, pediatrics, and general medicine. Our study showed that the number of uncited articles represents approximately 22 % which is considered high. However, the number of citations of any article varies from time to time and from one journal to another. Therefore, comparison of uncited articles from one subject to another might not be of great benefit.

Our study has a few limitations related to search strategy and methodology [12, 15, 30, 32, 80, 81]. For example, our study did not include articles published in non-Scopus database. However, Scopus remain a reliable and large source for bibliometric studies in general. Another limitation in our study is the keywords strategy. False positive and false negative results could be obtained regardless of how accurate the search stagey was. However, with the manual check of more than 300 articles, we believed that false positive or negative results will be very marginal and could hardly affect the accuracy of the results. Furthermore, the use of the keywords in title search instead of title/abstract/keywords would minimize false positive and negative articles and keep non-relevant articles in the minimum tolerable number. To the best of our knowledge, this is the first bibliometric study on probiotics in general and in pediatric in specific.

## Conclusions

The results of our study showed the following characteristics regarding probiotic publications: there is a growing interest in this topic as seen by the linear increase in the number of publications with time; there is a dominant leadership for Finland and USA in pediatric probiotic publications; there is a wide variety of journal names in which probiotics research is published; there is a great focus on clinical therapeutic application of probiotics as demonstrated by the title of hot articles in the field; and there is a wide variation in inter-country collaboration in probiotic research among the top leadership countries and there is a common trend toward domestic different countries.

## Abbreviations

AAD, antibiotic-associated diarrhea; FDA, food and drug administration; GDP, gross domestic product; h-index, the Hirsch index; IBD, inflammatory bowel disease; IBS, irritable bowel syndrome; IF, impact factor; IRB, institutional review board; NEC, necrotizing enterocolitis; SCR, standard competition ranking; USA The Unite States of America
